# Risk factors and predictive modeling for occult endometrial cancer in women with atypical hyperplasia: a retrospective study

**DOI:** 10.1007/s00404-026-08329-y

**Published:** 2026-01-29

**Authors:** Keren Wolff, Dina Gumin, Raneen Abu Shqara, Avishalom Sharon, Inshirah Sgayer, Lior Lowenstein, Ala Aiob

**Affiliations:** 1 Azrieli Faculty of Medicine, Bar Ilan University, Safed, Israel; 2 Department of Obstetrics and Gynecology, Galilee Medical Center, 89 Nahariya-Cabri, Nahariya, Israel

**Keywords:** Occult carcinoma, Sentinel lymph node biopsy, Predictive model, Hyperlipidemia

## Abstract

**Purpose:**

Atypical endometrial hyperplasia (AEH) is a known precursor to endometrioid endometrial carcinoma. However, occult carcinoma may already be present at diagnosis, complicating surgical planning. Accurate preoperative risk stratification is crucial, especially for guiding the selective use of sentinel lymph node biopsy. This study aimed to identify predictors of occult carcinoma and develop a model to estimate the risk of malignancy.

**Methods:**

We conducted a retrospective case–control study of 101 women diagnosed with AEH who underwent hysterectomy between 2010 and 2024 at Galilee Medical Center. Clinical, metabolic, and imaging data were extracted. Patients were stratified based on the final pathology into two groups: those with occult carcinoma and those with AEH only. Multivariable logistic regression was employed to identify independent predictors and construct a predictive model.

**Results:**

Occult endometrial carcinoma was identified in 37 women (36.6%). Women with occult endometrial carcinoma were older and more likely to present with postmenopausal bleeding. Occult carcinoma was more frequently detected after Pipelle biopsy than after hysteroscopy or dilation and curettage (43.2% vs. 17.2%). In multivariable analysis, Pipelle biopsy (OR 4.68), hyperlipidemia (OR 5.86), obesity (OR 2.97), and increasing age (OR 1.07 per year) were independently associated with occult carcinoma. A predictive model estimated individual risk ranging from 5.6% to 95.0% according to accumulation of risk factors.

**Conclusion:**

Older age, biopsy method, obesity, hyperlipidemia, and bleeding presentation are independently associated with an occult endometrial carcinoma in women with atypical endometrial hyperplasia. The proposed model may support preoperative risk stratification and counseling, but it requires external validation before clinical use, including decisions regarding sentinel lymph node biopsy.

**Supplementary Information:**

The online version contains supplementary material available at 10.1007/s00404-026-08329-y.

## What does this study add to the clinical work

**Table:** 

Occult endometrial carcinoma is found in over one-third of women with atypical endometrial hyperplasia. Factors, such as older age, biopsy method, obesity, hyperlipidemia, and postmenopausal bleeding, are independently associated with the presence of carcinoma. A predictive model that includes these factors may aid in personalized preoperative risk assessment and patient counseling; however, external validation and evaluation of its clinical utility are necessary before it can guide surgical decisions, including the selective use of sentinel lymph node biopsy.	

## Introduction

Endometrial cancer is the sixth most common cancer among women worldwide, with the highest incidence reported in developed regions, such as the United States and Europe [1]. Its incidence has continued to increase over recent decades, mainly due to rising obesity rates and changing reproductive patterns [2, 3]. Although there is no population-based screening program, endometrial hyperplasia is widely recognized as a precursor lesion, especially for endometrioid carcinoma, the most common histologic subtype [4]. Endometrial hyperplasia is characterized by excessive proliferation of endometrial glands relative to the stroma and has an estimated incidence of 133 cases per 100,000 woman-years in the United States [5]. It most commonly presents with abnormal uterine bleeding and is a well-established risk factor for endometrial carcinoma [6, 7]. According to the World Health Organization (WHO), endometrial hyperplasia is classified as atypical or non-atypical based on the presence of cytologic atypia [8]. Diagnosing endometrial carcinoma shortly after hyperplasia identification—particularly within the first few months—often indicates a pre-existing but initially undetected cancer, possibly due to sampling limitations or diagnostic misinterpretation at biopsy [9–11]. Previous studies consistently report that about 30–50% of women diagnosed with atypical endometrial hyperplasia (AEH) have concurrent endometrial carcinoma at hysterectomy, with systematic reviews and prospective studies showing positive predictive values of 37–43% [10, 12]. Accurate preoperative assessment of occult carcinoma risk in women with AEH is therefore crucial for guiding surgical planning, referral to gynecologic oncology, and considering sentinel lymph node biopsy [13]. However, there is no consensus on the routine use of sentinel lymph node biopsy in this population since lymph node involvement appears uncommon and existing data are conflicting [13]. Additionally, most previous studies have not systematically evaluated clinical, imaging, or metabolic predictors that could identify women with AEH at the highest risk of concurrent cancer. We hypothesized that a predictive model combining key clinical, imaging, and metabolic variables could improve personalized risk stratification and support more tailored surgical decisions. Such an approach might enable selective use of sentinel lymph node biopsy, reducing overtreatment in low-risk patients while ensuring appropriate staging in those at a higher risk. Finding reliable predictors of occult carcinoma remains an important unmet need for optimizing management strategies for women with AEH [14]. Therefore, the primary objective of this study was to identify factors associated with occult endometrial carcinoma in women diagnosed with AEH and develop a predictive model estimating the probability of concurrent malignancy. This model is intended to support preoperative risk stratification and clinical counseling, especially concerning surgical planning and staging decisions.

## Materials and methods

This was a retrospective, single-center case–control study conducted in the Department of Obstetrics and Gynecology to assess the risk factors and the probability of occult endometrial carcinoma in women diagnosed with AEH. This study was approved by the Galilee Medical Center Institutional Review Board (IRB number: NHR-0070-24), and informed consent was waived due to its retrospective nature.

This study included all women who underwent a hysterectomy between January 2010 and May 2024 following a histological diagnosis of AEH. At the institution, women diagnosed with AEH based on endometrial sampling, such as Pipelle biopsy, dilation and curettage, or hysteroscopy, are typically referred for hysterectomy, with or without bilateral salpingo-oophorectomy, within two months of diagnosis. AEH was diagnosed using the 2014 WHO criteria, with EIN terminology applied in some cases. Because both systems are managed equivalently at our institution, they were analyzed as a single diagnostic category. Pathologists were not formally blinded to clinical information at the time of hysterectomy specimen evaluation, reflecting routine real-world diagnostic practice. Occult endometrial carcinoma was defined as carcinoma found on final hysterectomy pathology after a preoperative diagnosis of AEH.

Women were identified by reviewing medical records and pathology reports from the hospital’s archives. Cases were also included if the initial diagnosis was made outside the hospital (for example, in community clinics or other hospitals).

Still, surgery and final pathology were completed at Galilee Medical Center. All hysterectomy specimens were reviewed at our institution by gynecologic pathologists, and AEH and carcinoma were diagnosed according to institutional practice standards. For biopsies performed externally, pathology reports were accepted without mandatory central slide review.

All patients underwent a standardized preoperative assessment, which included a medical history, physical examination, laboratory tests (complete blood count and serum chemistry), and a transvaginal ultrasound (TVS) to assess uterine size and endometrial thickness. Hyperlipidemia was defined as a documented diagnosis of dyslipidemia and/or current use of lipid-lowering medication at the time of preoperative assessment. Obesity was defined as body mass index (BMI) ≥ 30 kg/m^2^. Pipelle biopsies were performed in the outpatient clinic by senior gynecologists or by residents under direct supervision, without anesthesia.

Inclusion criteria were women aged from 18 to 80 years, a histologically confirmed diagnosis of AEH on initial endometrial sampling, and who underwent hysterectomy within two months of the initial diagnosis. Exclusion criteria included receipt of progestin therapy before hysterectomy and hysterectomy performed more than two months after the initial diagnosis.

Patients were divided into two groups based on final histopathological results from hysterectomy specimens: the case group included women with a final diagnosis of endometrial carcinoma. In contrast, the control group consisted of women with a final diagnosis of endometrial hyperplasia without carcinoma.

## Statistical analysis

Categorical variables were summarized as frequencies and percentages. Continuous variables were assessed for normality through visual inspection of histograms and are presented as mean ± standard deviation for normally distributed data or median (range) for non-normally distributed data. For inferential analysis, categorical variables were compared between groups using chi-square test or Fisher’s exact test as appropriate (expected cell count < 5). Continuous variables were compared using the independent samples t test for normally distributed data or Mann–Whitney U test for non-normally distributed data. All statistical tests were two-sided, with a *p* value < 0.05 indicating statistical significance.

Multivariable logistic regression analysis was performed to identify independent predictors of occult endometrial carcinoma, with the presence of carcinoma on final hysterectomy pathology as the dependent variable. Clinically relevant variables and those associated with the outcome in univariate analysis were included in the multivariable model.

Multicollinearity among metabolic variables, including obesity, diabetes mellitus, and hyperlipidemia, was assessed prior to model construction using variance inflation factors (VIFs), with no evidence of significant collinearity identified. Missing data were infrequent, accounting for less than 5% of observations across all variables; therefore, analyses were performed using complete case data.

A multivariable logistic regression model was developed to estimate the probability of occult endometrial carcinoma. The final predictive model is described by the following logistic regression equation: logit(p) = *β*₀ + *β*₁(age) + *β*₂(biopsy method) + *β*₃(hyperlipidemia) + *β*₄(obesity) + *β*₅(postmenopausal bleeding), where *p* represents the probability of occult endometrial carcinoma. The predicted probability was calculated as *p* = 1/(1 + e^(−logit(*p*))). Statistical analysis was performed using IBM SPSS Statistics software, version 25.0 (IBM Corp., Armonk, NY, USA).

## Results

**Fig. 1:**
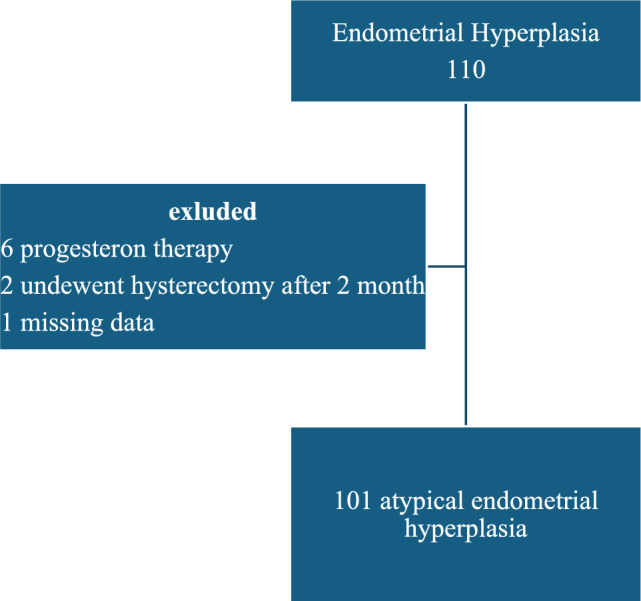
Flowchart of study population selection, including exclusions and reasons for exclusion

This study initially identified 110 women diagnosed with AEH who underwent hysterectomy between 2010 and 2024. After applying the exclusion criteria, 101 women were included in the final analysis. Figure 1 presents a flowchart of the study population selection, including exclusions and reasons for exclusion. The mean age was 55.3 ± 10.9 years. Endometrial sampling was performed via Pipelle biopsy in 27 women (26.7%) and through hysteroscopy or D&C in 74 women (73.3%). The median BMI was 30.5 (21.1–51.0 kg/m^2^). Regarding clinical presentation, 14 women (14.4%) were asymptomatic, 46 (47.4%) presented with abnormal uterine bleeding (AUB), and 37 (38.1%) with postmenopausal bleeding (PMB). (Table 1) Following final histopathological examination of the hysterectomy specimens, 37 women (36.6%) were found to have occult endometrial carcinoma, while 64 women (63.4%) had hyperplasia without malignancy.

**Table 1:** Baseline clinical, biopsy, and imaging characteristics of women diagnosed with atypical endometrial hyperplasia

(*N* = 101)	
Age, Median (range)	53.0 (34.5–88.0)
BMI, Median (range)	30 (22–51)
Smoker, *n* (%)	20 (23%)
Hypertension, *n* (%)	46 (48.4%)
Diabetes mellitus, *n* (%)	36 (38.3%)
Hyperlipidemia, *n* (%)	26 (27.7%)
Obesity, *n* (%)	48 (51.1%)
Hypothyroidism, *n* (%)	8 (8.5%)
Family history of cancer, *n* (%)	29 (32.9%)
Parity, Median (range)	3 (0–11)
Cesarean sections, Median (range)	0 (0–2)
Abortions, Median (range)	0 (0–7)
Symptoms at administration *n* (%)	
Vaginal bleeding	64 (74.4%)
Pain	1 (1.1%)
Asymptomatic	6 (6.9%)
TVS findings *n* (%)	
Endometrial thickness 1–4 mm	32 (39.5%)
Endometrial thickness 5–10 mm	16 (19.8%)
Endometrial thickness 11+ mm	33 (40.7%)
Irregular endometrial layer	24 (33.8%)
Polyps	4 (4%)
Occult cancer by final histology *n* (%)	37 (36.6%)

*BMI* body mass index, *TVS* transvaginal sonography

Of the 37 women (36.6%) diagnosed with occult endometrial carcinoma, most tumors were low-grade endometrioid. Higher-grade disease was uncommon, with grade 2 in 5.4% and grade 3 in 16.2% of cases. Lymphovascular space invasion was present in 8.1%, deep myometrial invasion (> 50%) in 29.7%, and pelvic lymph node involvement in 5.4%. Most cancers were early stage, with FIGO stage IB in 19.4%, while advanced disease (FIGO stages II–IIIC) occurred in 11.2% of patients (Table 2).

**Table 2:** Characteristics of the occult endometrial cancer

(*N* = 37)	
Endometrioid cancer grade 1, *n* (%)	27 (73%)
Endometrioid cancer grade 2, *n* (%)	2 (5.4%)
Endometrioid cancer grade 3, *n* (%)	6 (16.2%)
Malignant cell in cytology, *n* (%)	2 (5.4%)
Lymph vascular space invasion, *n* (%)	3 (8.1%)
Myometrial invasion > 50%, *n* (%)	11 (29.7%)
Pelvic lymph node involvement, n(%)	2 (5.4%)
Stage 1A, *n* (%)	26 (72.2)
Stage 1B, *n* (%)	7 (19.4%)
Stage 2, *n* (%)	2 (5.6%)
Stage 3c, *n* (%)	2 (5.6%)

Staging according to FIGO 2009

Women with occult carcinoma were significantly older than those without cancer (61.5 ± 11.5 vs. 51.7 ± 8.7 years, *p* < 0.001). PMB was more frequently reported in the occult cancer group (54.1% vs. 10%, *p* = 0.001), while asymptomatic presentation occurred exclusively in the non-cancer group (10.9% vs. 0%, *p* = 0.081). Occult carcinoma was significantly more common in women who underwent Pipelle biopsy for initial diagnosis (43.2% vs. 17.2%) and less common among those who had D&C (*p* = 0.007).

Metabolic comorbidities were also significantly associated with occult malignancy: obesity (63.9% vs. 43.1%, *p* = 0.04), diabetes mellitus (52.8% vs. 29.3%, *p* = 0.03), and hyperlipidemia (42.2% vs. 15.5%, *p* = 0.001) were all more prevalent in the cancer group. No significant differences were observed between groups in neutrophil-to-lymphocyte ratio (NLR), endometrial thickness on ultrasound, or presence of endometrial polyps.** (**Table 3**).**

**Table 3:** Comparison of clinical, biopsy, imaging, and laboratory variables between women with and without occult endometrial carcinoma

*N* = 101	No malignancy 64 (63.4%)	Malignancy 37 (36.6%)	*P*-value
Age, Median (range)	51.0 (34–75)	61 (37–88)	< 0.001^+^
Parity, Median (range)	3 (0–11)	2 (0–10)	0.056^++^
Abortions, Median (range)	0 (0–7)	0 (0–3)	0.950^++^
Biopsy, *n* (%)			
Pipelle	11 (17.2%)	16 (43.2%)	0.007^*^
Hysteroscopy	26 (40.6%)	14 (37.8%)	
Dilation and curettage	27 (42.2%)	7 (18.9%)	
Patient history			
BMI, Median (range)	30.1 (21–51)	31.6 (22–42)	0.446^+^
History of malignancy in the family	16 (29.1%)	13(39.4%)	0.204^**^
Smoking	11 (20.8%)	9 (26.5%)	0.606^*^
Any disease	43 (63.8%)	35 (94.6%)	0.001^*^
Hypertension	24 (41.4%)	22 (59.5%)	0.065^++^
Diabetes mellitus	17 (29.3%)	19 (52.8%)	0.030^*^
Hyperlipidemia	9 (15.5%)	17 (47.2%)	0.001^*^
Obesity	25 (43.1%)	23 (63.9%)	0.040^++^
Hypothyroidism	6 (10.3%)	2 (5.6%)	0.706^**^
Presenting symptom, *n* (%)			
Asymptomatic	6 (10.9%)	0 (0%)	0.081^**^
Symptoms unrelated to vaginal bleeding	6 (10.0%)	8 (21.6%)	0.001^*^
AUB	37 (61.7%)	9 (24.3%)	0.001^*^
PMB	17 (28.3%)	20 (54.1%)	0.001^*^
Endometrial thickness by TVS at diagnosis of atypical endometrial hyperplasia, *n* (%)			
1–4 mm	18 (37.5%)	14 (42.4%)	0.622^++^
5–10 mm	13 (27.1%)	3 (9.1%)	
11>	17 (35.4%)	16 (48.5%)	
Polyp by TVS	2 (4.7%)	1 (2.7%)	1.000^**^
Laboratory findings			
NLR, mean (± sd)	7.2 (± 6.8)	8.2(± 8.4)	0.353^++^

*BMI* body mass index, *AUB* abnormal uterine bleeding, *PMB* postmenopausal bleeding, *TVS* transvaginal sonography, *NLR* neutrophil-to-lymphocyte ratio

^*^Chi-square

^**^Fisher’s test

^+^Independent samples T test

^++^Mann

Multivariable logistic regression identified several independent predictors of occult endometrial carcinoma (Table 3). Diagnosis by Pipelle biopsy was associated with a significantly higher risk compared with hysteroscopy or dilation and curettage (OR 4.68; 95% CI 1.33–16.48; *p* = 0.016). Hyperlipidemia showed a strong independent association with malignancy (OR 5.86; 95% CI 1.65–20.79; *p* = 0.006). Obesity was associated with a moderate increase in the risk of occult carcinoma (OR 2.97; 95% CI 0.92–9.60; *p* = 0.069), while increasing age was associated with a modest but statistically significant increase in risk (OR 1.07 per year; 95% CI 1.01–1.14; *p* = 0.023). Based on these findings, a multivariable predictive model was developed that includes biopsy method, hyperlipidemia, obesity, and bleeding pattern to estimate personalized probabilities of occult endometrial carcinoma. Within this model, Pipelle biopsy, hyperlipidemia, obesity, and postmenopausal bleeding each play a meaningful role in risk prediction, with full effect estimates provided in Table 4.

**Table 4:** Multivariable logistic regression predictors of occult endometrial carcinoma

Predictor	Odds ratio (OR)	95% CI (Lower–upper)	*P* value
Pipelle vs. hysteroscopy/D&C	3.80	1.21–11.93	0.022
Hyperlipidemia	6.38	2.02–20.15	0.002
Obesity	3.33	1.14–9.73	0.028
PMB vs. AUB	3.95	1.40–11.09	0.009

AUB *abnormal uterine bleeding*, *PMB* postmenopausal bleeding, *D&C* dilation and curettage

A predictive model calculated personalized probabilities of occult endometrial carcinoma in various clinical scenarios. The risk rose progressively with the addition of risk factors. Women with no risk factors, such as diagnosis via hysteroscopy or dilation and curettage, no hyperlipidemia or obesity, and no postmenopausal bleeding, had an estimated risk of 5.6%. Conversely, women presenting all four risk factors, Pipelle biopsy, hyperlipidemia, obesity, and postmenopausal bleeding, had a predicted risk of 95.0% (Table 5). Intermediate estimates ranged from about 16% to 85%, depending on the combination and number of risk factors (Supplemental Table 1).

**Table 5:** Predicted probability of occult endometrial carcinoma by number of risk factors present

Number of risk factors present	Risk factor combination	Predicted probability (%)
0 (reference)	Hysteroscopy/D&C, no hyperlipidemia, no obesity, no PMB	5.6
1	Any one of: obesity **or** hyperlipidemia **or** PMB **or** Pipelle biopsy	16–27
2	Any two risk factors present	43–59
3	Any three risk factors present	75–85
4	Pipelle biopsy + hyperlipidemia + obesity + PMB	95

Risk factors included biopsy method (Pipelle vs hysteroscopy/D&C), hyperlipidemia, obesity (BMI ≥ 30 kg/m^2^), and postmenopausal bleeding (PMB). Predicted probabilities were derived from the multivariable logistic regression model

**Fig. 2:**
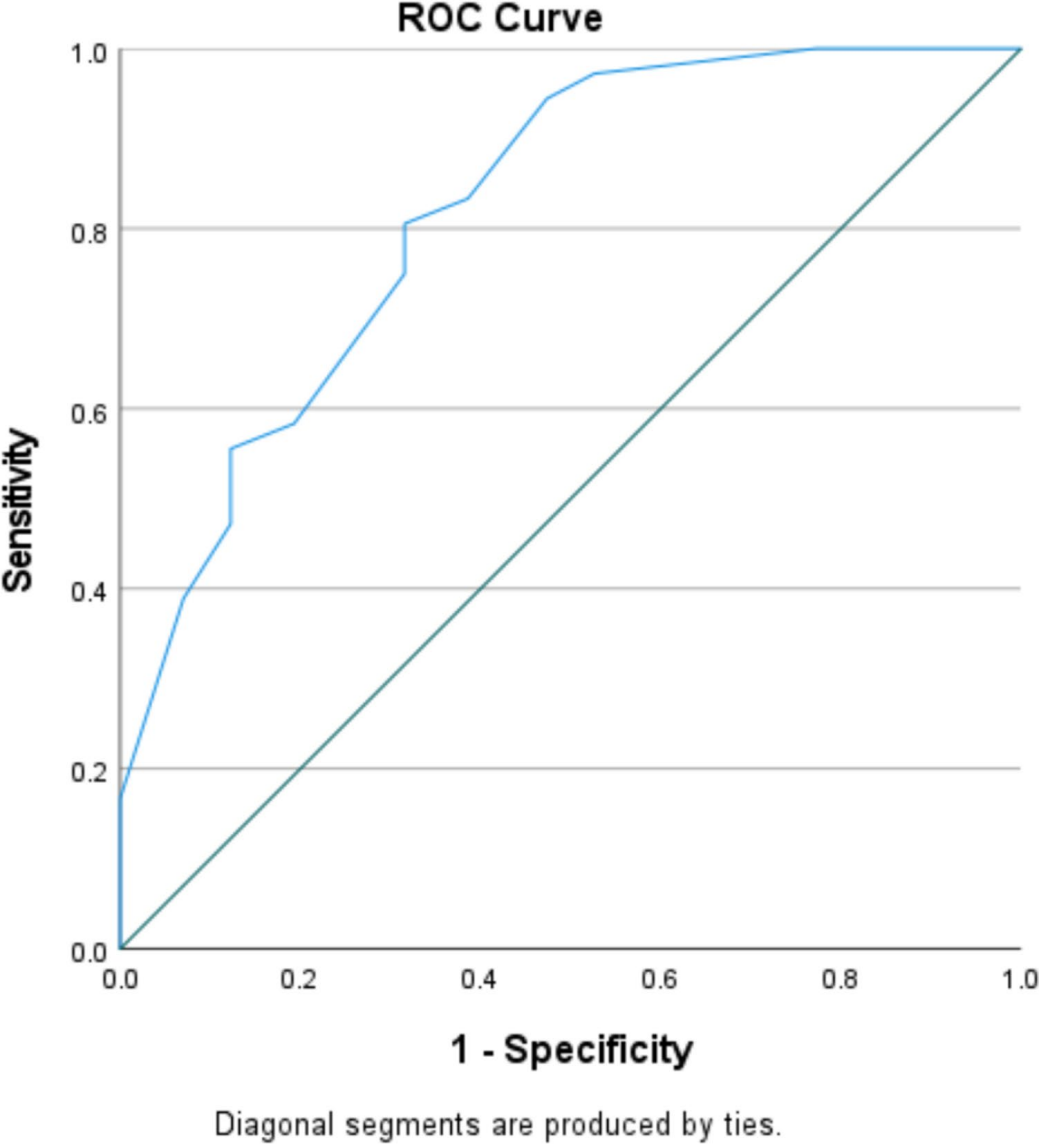
Receiver operating characteristic (ROC) curve of the predictive model for occult endometrial carcinoma in women with atypical endometrial hyperplasia

The predictive model showed strong discriminative ability, with an area under the receiver operating characteristic (ROC) curve of 0.82 (95% confidence interval [CI] 0.74–0.90; *p* < 0.001; Fig. 2). Model calibration was evaluated using the Hosmer–Lemeshow goodness-of-fit test and indicated good agreement between predicted and observed outcomes (*p* = 0.418). Internal validation with bootstrap resampling (1000 samples) confirmed the stability of the regression coefficients; although the intercept varied slightly, the discriminative performance remained consistent (AUC = 0.82, 95% CI 0.74–0.90), demonstrating robust model performance.

## Discussion

This study identified several clinical and metabolic predictors of occult endometrial carcinoma in women diagnosed preoperatively with AEH. Specifically, advanced age, obesity, hyperlipidemia, and the method of endometrial sampling emerged as significant risk factors. These findings align with and add detail to previous studies, reinforcing the need for personalized risk assessment when determining surgical management and sentinel lymph node evaluation. We found a 36.6% prevalence of occult carcinoma among women with AEH who underwent hysterectomy. This rate is consistent with previously reported estimates, which range from 30 to 50%. For example, Doherty et al.’s systematic review and meta-analysis reported a pooled prevalence of 32.6% (95% CI: 24.1–42.4%) [14]. In comparison, Vetter et al. observed a rate of 48.5% in women with endometrial intraepithelial neoplasia or complex atypical hyperplasia at the time of surgery [15]. These findings highlight the diagnostic limitations of preoperative endometrial sampling and underscore the value of enhanced pre-surgical assessment tools.

Age was a strong predictor of concurrent carcinoma in our cohort, with risk increasing steadily with each passing year. This corresponds with data from Matsuo et al., who found that women aged 40–59 and ≥ 60 years had odds ratios (ORs) of 3.07 and 6.65, respectively, compared to younger patients [16]. Similar links were reported by Çaltek et al. (OR 3.94 for women ≥ 50 years) and Rajadurai et al., who observed that patients with carcinoma were notably older than those without (average age 60.2 vs. 55.5 years) [17, 18]. Obesity also showed a significant and independent association with occult malignancy, especially at a BMI of 35 kg/m^2^ or higher. These findings align with those of Matsuo et al. (OR 2.32) and Çaltek et al., who also identified high BMI as a significant risk factor [16, 17]. These observations match the well-known connection between adiposity, chronic estrogen exposure, and the development of endometrial cancer. Hyperlipidemia was independently associated with an increased risk of occult carcinoma in our study, suggesting a potential role for lipid dysregulation in endometrial carcinogenesis, particularly within the broader context of metabolic syndrome. Although hyperlipidemia has been less extensively studied than obesity or diabetes, large population-based cohorts and meta-analysis have demonstrated a modest but consistent association between dyslipidemia and endometrial cancer risk (OR 1.15–1.25) [19, 20]. Proposed biologic mechanisms include altered lipid metabolism, chronic low-grade inflammation, oxidative stress, and interactions between lipid pathways and estrogen signaling, which may promote endometrial proliferation and tumor progression. Our findings extend this literature by suggesting that hyperlipidemia may also be relevant in the premalignant setting of AEH and should be considered in future risk stratification models and counseling.

Diabetes mellitus was more prevalent among women with occult carcinoma, though it did not achieve statistical significance in multivariate analysis. Nonetheless, previous research has identified diabetes as a significant independent risk factor (e.g., OR 2.51), even after adjusting for age and BMI [16, 21]. These findings highlight the importance of assessing metabolic health comprehensively when evaluating patients with AEH.

The method used for preoperative endometrial sampling significantly influences diagnostic accuracy. Pipelle biopsy has a higher risk of missing malignancies compared to D&C or hysteroscopic biopsy. This aligns with previous research, which shows that Pipelle has lower sensitivity for detecting focal or early-stage lesions. Meta-analysis confirms this limitation, with Pipelle showing lower sensitivity (0.77, 95% CI: 0.57–0.90) compared to D&C (0.88, 95% CI: 0.28–0.99) although both methods have similarly high specificity (> 98%) [20, 22, 23]. These results suggest a more targeted approach to choosing the biopsy method, especially for patients at higher clinical risk. It is important to note that the association between Pipelle biopsy and occult carcinoma should be interpreted cautiously as biopsy sensitivity was not directly assessed and the result may be influenced by selection bias or residual confounding rather than an inherent flaw of the method.

In the multivariable analysis, hyperlipidemia, obesity, use of Pipelle biopsy, and PMB presentation were independently linked to a significantly higher risk of occult carcinoma in women with AEH. Our risk calculator, which combines these factors, offers individualized risk estimates ranging from 5.6% to 94.9%, supporting accurate and shared decision-making. This predictive model may serve as a practical tool for personalized risk assessment, aiding surgical decisions, such as selective use of sentinel lymph node biopsy and timely referral to gynecologic oncology. Importantly, these predicted probabilities should be viewed as illustrative estimates rather than strict thresholds. Due to the retrospective design, single-center cohort, and lack of external validation, the model is intended to assist with preoperative risk assessment and patient counseling, not to directly guide clinical decisions, such as selective sentinel lymph node biopsy.

For patients with a high predicted risk, especially those with multiple risk factors, the model recommends prioritizing hysterectomy with comprehensive staging, including sentinel lymph node biopsy, consistent with ACOG guidelines to rule out concurrent carcinoma before conservative treatment. Obesity and uterine factors such as cavity distortion or technical sampling limitations may reduce the adequacy of the Pipelle biopsy, and this should be considered when interpreting negative results. Conversely, low-risk patients may be suitable for medical management or less invasive procedures to help avoid overtreatment. In ambiguous cases, the model may suggest further testing, such as hysteroscopic-guided biopsy, which ACOG supports for its greater accuracy in detecting focal lesions. These findings highlight the importance of including metabolic risk factors, like hyperlipidemia, in gynecologic oncology risk assessments and counseling [24].

A key strength of our study is the development of a practical, clinically applicable predictive model using readily available variables, including age, BMI, hyperlipidemia, and biopsy method, to estimate the risk of occult endometrial carcinoma. Our findings are based on a well-defined cohort of women with histologically confirmed AEH, enhancing the reliability of risk estimates. Additionally, the inclusion of metabolic comorbidities, particularly hyperlipidemia, addresses under-recognized but clinically relevant factors in risk stratification.

Several limitations of this study should be acknowledged. The retrospective, single-center design and inclusion of only women who underwent hysterectomy likely enriched the cohort for higher-risk patients. Decisions regarding surgical versus conservative management may have been influenced by unmeasured clinical factors, patient characteristics, and evolving provider practice patterns over the study period (2010–2024), potentially leading to overestimation of malignancy risk when the model is applied to more conservatively managed populations. Data on lesion size and detailed radiologic findings were not available and may further improve predictive accuracy in future models. In addition, interobserver variability in diagnosing AEH was not systematically assessed, and external biopsy specimens were not consistently re-reviewed, which may introduce misclassification bias. Finally, although the model demonstrated good discriminative performance in this cohort, external validation in larger, multicenter populations is required before clinical implementation. Temporal changes in diagnostic and surgical practices, including increased use of hysteroscopy, may have contributed to heterogeneity that could not be formally evaluated.

In conclusion, our study highlights the significant impact of age, obesity, hyperlipidemia, and biopsy method on the risk of occult endometrial carcinoma in women with AEH. We propose a simple, accurate predictive model that may support individualized surgical planning. This approach may improve oncologic outcomes while minimizing overtreatment, aligning with current recommendations for risk-adapted care in gynecologic oncology. However, external validation and evaluation of its clinical utility are necessary before it can guide surgical decisions, including the selective use of sentinel lymph node biopsy.

## Supplementary Information

Below is the link to the electronic supplementary material.

Supplementary file1 (DOCX 15 KB)

## Data Availability

No datasets were generated or analysed during the current study.
