# Supplementary material for: Risk factors and predictive modeling for occult endometrial cancer in women with atypical hyperplasia: a retrospective study
Source: Arch Gynecol Obstet. 2026 Jan 29;313(1):67. doi: 10.1007/s00404-026-08329-y (PMC12855440; doi:10.1007/s00404-026-08329-y)
Supplement: Supplementary file 1 — Supplementary file1 (DOCX 15 KB) [file 404_2026_8329_MOESM1_ESM.docx]

**Supplemental Table 1. Predicted Probability of Cancer Based on Risk Factor Combinations**

| **Biopsy Method** | **Hyperlipidemia** | **Obesity** | **PMB** | **Predicted Probability** |
| --- | --- | --- | --- | --- |
| Hysteroscopy/D&C | No | No | No | 0.05579 |
| Hysteroscopy/D&C | No | Yes | No | 0.16446 |
| Pipelle biopsy | No | No | No | 0.18353 |
| Hysteroscopy/D&C | No | No | yes | 0.18907 |
| Hysteroscopy/D&C | Yes | No | No | 0.27378 |
| Pipelle biopsy | No | Yes | No | 0.42816 |
| Hysteroscopy/D&C | No | Yes | Yes | 0.43714 |
| Pipelle biopsy | No | No | yes | 0.47004 |
| Hysteroscopy/D&C | Yes | Yes | No | 0.55669 |
| Pipelle biopsy | Yes | No | No | 0.58917 |
| Hysteroscopy/D&C | Yes | No | yes | 0.59799 |
| Pipelle biopsy | No | Yes | yes | 0.74711 |
| Hysteroscopy/D&C | Yes | Yes | yes | 0.83207 |
| Pipelle biopsy | Yes | No | yes | 0.84982 |
| Pipelle biopsy | Yes | Yes | yes | 0.94962 |

*PMB, postmenopausal bleeding;D&C, dilation and curettage*
